# *Galleria mellonella* as an Invertebrate Model for Studying Fungal Infections

**DOI:** 10.3390/jof11020157

**Published:** 2025-02-18

**Authors:** Gabriel Davi Marena, Luciana Thomaz, Joshua Daniel Nosanchuk, Carlos Pelleschi Taborda

**Affiliations:** 1 Institute of Biomedical Science, Department of Microbiology, University of São Paulo (ICB II—USP), São Paulo 05508-900, Brazil; lucithomaz2016@gmail.com; 2 Departments of Medicine and Microbiology and Immunology, Albert Einstein College of Medicine, Bronx, NY 10461, USA; josh.nosanchuk@einsteinmed.edu; 3 Laboratory of Medical Mycology, Institute of Tropical Medicine of São Paulo/LIM53, School of Medicine, University of São Paulo, São Paulo 05403-000, Brazil

**Keywords:** *Galleria mellonella*, virulence, fungal infection, antifungals, resistance, new therapies

## Abstract

The incidence of fungal infections continues to increase and one of the factors responsible for these high rates is the emergence of multi-resistant species, hospitalizations, inappropriate or prolonged use of medications, and pandemics, such as the ongoing HIV/AIDS pandemic. The recent pandemic caused by the severe acute respiratory syndrome virus (SARS-CoV-2) has led to a significant increase in fungal infections, especially systemic mycoses caused by opportunistic fungi. There is a growing and urgent need to better understand how these microorganisms cause infection and develop resistance as well as to develop new therapeutic strategies to combat the diverse diseases caused by fungi. Non-mammalian hosts are increasingly used as alternative models to study microbial infections. Due to their low cost, simplicity of care, conserved innate immunity and reduced ethical issues, the greater wax moth *Galleria mellonella* is an excellent model host for studying fungal infections and it is currently widely used to study fungal pathogenesis and develop innovative strategies to mitigate the mycoses studied. *G. mellonella* can grow at 37 °C, which is similar to the mammalian temperature, and the anatomy of the larvae allows researchers to easily deliver pathogens, biological products, compounds and drugs. The aim of this review is to describe how *G. mellonella* is being used as a model system to study fungal infections as well as the importance of this model in evaluating the antifungal profile of potential drug candidates or new therapies against fungi.

## 1. Introduction

Pathogenesis research and the development of novel therapeutics require robust pre-clinical modelling. Due to ethical considerations, researchers are striving to utilize non-mammalian species for translational work. *Galleria mellonella* larvae are a powerful tool for studying microbial infections and the antimicrobial profile of medicines, before proceeding with clinical trials in mammals, reinforcing the application of the principles of the 3Rs (replacement, reduction and refinement) involving animals [1]. The first formal ethical guidelines on the use of animals in experiments were defined by psychologist Marshall Hall in 1831. Later, in 1957, the concept of the 3Rs was presented at a Universities Federation for Animal Welfare (UFAW) Symposium [2]. Since then, alternative experimental models have been widely sought after and used to reduce the use of mammals [3]. In recent decades, the number of in vivo studies using insects has increased dramatically. In particular, *G. mellonella* have emerged as a leading non-mammalian model as their immune system presents characteristics similar to the immune response found in humans, such as innate defense, therefore, they can be called the “evolutionary roots of human innate immunity” [4].

The *G. mellonella* model readily allows for the evaluation of the activity and toxicity profile of diverse compounds in settings with and without infection by various microbes [5]. *G. mellonella* is known as a moth that “has never been stung by a bee” and develops into a caterpillar known as a “borer”, first described by Aristotle in c. 350 B.C. Also called the “wax moth”, *G. mellonella* belongs to the order Lepidoptera, family Pyralidae and subfamily Galleriinae [6].

Given these characteristics, many studies have been using this in vivo model for studies involving fungi, especially to evaluate the virulence profile of species or individual isolates and to assess potential antifungal candidates [7]. The larvae can be maintained at a temperature range from 25–37 °C, which includes the normal average human body temperature (37 °C), and this spectrum is essential to researchers who work with fungal pathogens [8].

Some advantages, such as ease of handling, low maintenance cost, minimal ethical restrictions, and the possibility of working with large quantities of larvae, are factors that contribute to the increase in research using *G. mellonella* models. Furthermore, some macroscopic changes can be easily detected in larvae, which allows for physiology studies [9]. This review provides updated information on the profile of fungal infection in *G. mellonella* models and discusses the potential antifungal action of promising candidates to combat the infection using this in vivo assay. Finally, Table 1 summarizes all studies discussed in this review.

## 2. Anatomic and Life Cycle of *G. mellonella*

**Figure 1:**
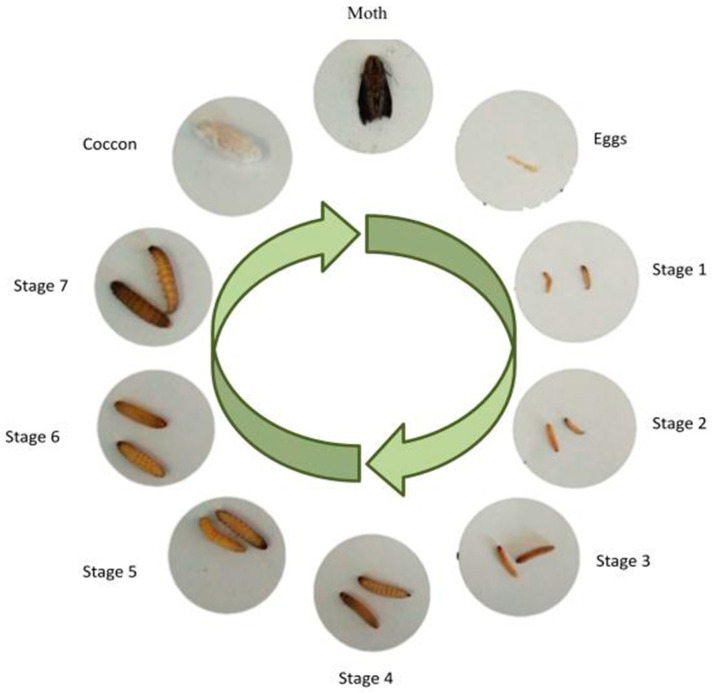
The life cycle of *G. mellonella.* Larvae were cultured at the University of São Paulo Department of Microbiology Laboratory of Dimorphic Fungal Pathogens (LFDP/ICB/USP) at 28.5 °C and fed with an artificial diet (pollen and honeycomb). The eggs are cream/white in color, clustered together and attached to a paper. Stage 1 are newly hatched larvae and evolve into Stage 2, weighing up to 0.05 g. From Stage 3 to Stage 6, larvae weigh between 0.05 and 0.2 g and are not used for testing (according to most published articles). Stage 6 larvae are ideal for testing, weighing more than 0.2 g. Furthermore, these larvae must present good motility and absence of melanin. At the end of the life cycle, the larvae form a cocoon, a hardened structure wrapped in webbing. Finally, after about 10–15 days, the cocoons hatch to release the moth (gray in color) to begin a new cycle with the acquisition of new eggs.

The life cycle of *G. mellonella* from eggs to moths can last approximately 8 to 13 weeks. This insect has four different stages in its life cycle: eggs, caterpillar, pre-pupa/pupa, and adult insect (moth). The eggs are from white to light pink, smooth and reticulate, and approximately 0.35 mm wide and 0.45 mm in length. The eggs hatch after 5 to 8 days. After developing into caterpillars, their sizes are defined as either small (0.5–10 mm), medium (10–20 mm), or large (>20 mm) [10,11]. The larval stage can last 6 to 7 weeks (28–32 °C), going through 8 to 10 molting stages, including silk production, ending with the formation of the cocoon (intermediate stage of pre-pupae). The cocoon is the penultimate stage of their cycle, presenting a dark red/or dark orange color. A pupa can take 1 to 8 weeks to transform into a moth, and this all occurs in the absence of food. Finally, in the last stage, the female moths are larger and darker in color than the male moths and have a forked proboscis and frontal projection labial palps. Females can lay around 50 to 150 eggs. Depending on environmental conditions, humidity, temperature, etc., the shelf life, of eggs to adult larvae, can be approximately 40 days [11]. Figure 1 represents the complete life cycle of *G. mellonella*.

**Figure 2:**
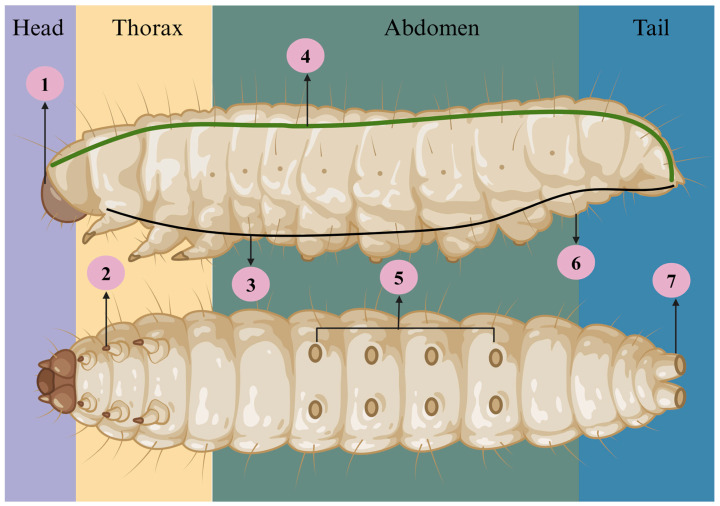
Anatomical regions of *G. mellonella.* (1): Head; (2): thoracic leg; (3): nervous system; (4): dorsal vessel; (5): abdomen prolegs; (6): protective outer tissue with amphiphilic characteristic; (7): anal proleg. Created in Biorender.com/y25x774.

**Figure 3:**
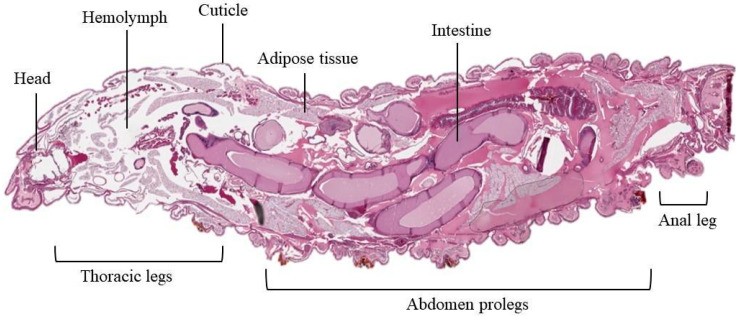
Using a slide viewer program, a cross-section of *G. mellonella* was selected for analysis in Philips IMS Scanners.

As represented in Figure 2 and Figure 3, the larva has four important anatomic regions, back (rostral or upper region), thorax, abdomen and tail (distal or lower region). The body of *G. mellonella* is composed of six thoracic legs (2), a hydrophobic outer layer (5), eight prolegs in the abdominal cavity (6), a distal leg (7, anal proleg), a dorsal vessel (4), and a nervous system (3) [12,13].

## 3. Immune System

*G. mellonella* has an innate immune system that has significant structural and functional similarities to that of mammals [14,15] and certain other insects, such as Lepidoptera [16]. Two important immune response mechanisms are triggered in *G. mellonella*: cellular and humoral responses. The cellular response is mediated by the presence of hemocytes that act through phagocytosis, nodulation and encapsulation of pathogens, while the humoral response is mediated by effector molecules, including reactive oxygen and nitrogen species (ROS and RNS), opsonins, antimicrobial peptides (AMPs), melanization mediated by phenoloxidase activation, and hemolymph coagulation [17].

The defense cells responsible for phagocytosis are called hemocytes, which are found free in the hemolymph or tissues and have a function analogous to the cells of mammals. Pathogen recognition occurs in two ways: direct interaction between the hemocyte surface receptor (pathogen recognition receptor—PRR) with pathogen surface molecules (pathogen-associated molecules (PAMPs). PAMPs are preserved microbial molecules, including lipopolysaccharides, peptidoglycans, lipoteichoic acids, and glucans. The second way is by indirect recognition of humoral receptors that interact as opsonins to the microorganism [1].

There are five main hemocytes—types classified according to their microscopic appearance, being spherulocytes (SP), granulocytes or granular cells (GR), prohemocytes (PR), plasmocytes (PL), and oenocytoides (OE). Granulocyte-type hemocytes are the cells most present in *G. mellonella* and are the main phagocytes [3]. Plasmocytes are also involved in phagocytosis. Prohemocytes are stem cells that differentiate into other hemocytes. Oenocytoids and spherulocytes are involved in the phenoloxidase cascade and secretion of cellular components, respectively. However, a recent study reports the existence of a six hemocyte, the coagulocyte, which is responsible for the healing process [18].

**Figure 4:**
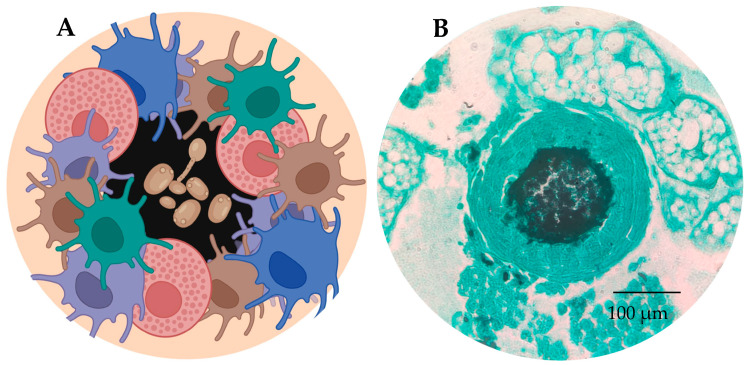
Aggregates of *G. mellonella* hemocytes form a granuloma structure in response to microbial invaders. (**A**): Schematic of a granuloma created in Biorender.com/r06i065; (**B**): granuloma formed in response to *Candidozyma auris* (taken from a study involving virulence analysis of *Candidozyma auris*) stained with Grocott gomori, scale bar 100 µm.

Another important mechanism of defense against microrganisms is the formation of granulomas or nodules (Figure 4). These granulomas are of variable size and are formed by clusters of hemocytes that attach around a pathogen to prevent the invading microbe’s dissemination. The result is a capsule that often includes deposited melanin [1,19]. Different substances such as coagulation factors, immune complexes and immunological enzymes are released during nodulation, leading to the death of the encompassed pathogen [20]. The production of melanization (yellow/orange/brown) indicates a strong immunological reaction to the pathogen [21].

The humoral response of *G. mellonella* is mediated by opsonins, antimicrobial peptides (AMPs) lysozymes and the phenoloxidase cascade. Opsonins are proteins produced in the hemolymph responsible for recognizing microbial components present in the cell wall. Among the main molecules recognized by opsonins are glucans, peptidoglycan and lipopolysaccharides [22]. Apolipophorin-III, for example, interacts with hydrophobic structures, such as lipoproteins and lipoteichoic acid. In fungi, interaction with glucans has been reported, increasing the encapsulation of the fungal structure [23].

The larva produces different AMPs depending on the type of microorganism. Eighteen peptides with different biochemical and antimicrobial properties have been identified in *G. mellonella* [24]. Cecropin-like peptide D, anionic peptide 2 [24], Gm defensin-like peptide, Gm proline-rich peptide 2, Gm anionic peptides 1 and 2, Gm apolipophoricin [25], lysozyme, Gm anionic peptide 1 (P1-like), cecropins, moricin-like peptides, gloverin, Gm proline-rich peptide 1, Gm proline-rich peptide 2, Gm anionic peptide 2, gallerimycin, galiomycin, and others are examples of AMPs [26].

**Figure 5:**
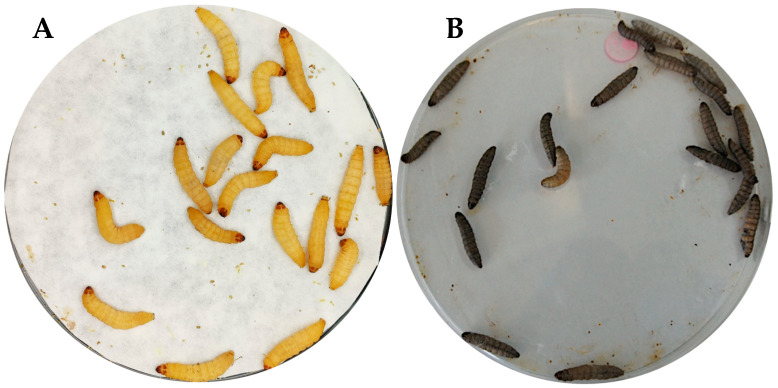
*G. mellonella* produces melanin in response to microbial invasion. (**A**): Non-infected group treated with phosphate-buffered saline; (**B**): group infected with *Candida parapsilosis* and presence of melanization.

Melanin formation is part of the larva’s humoral response mechanism; however, because it is toxic, its production is highly regulated. Production occurs through the activation of the prophenoloxidase cascade after infection, which activates serine proteases [27] release by hemocytes—mainly oenocytoides, but also granulocytes and plasmatocytes [28]. Melanization begins after pathogen recognition, followed by phagocytosis, stimulating the phenoloxidase pathway. The serine protease cascade acts to cleave the zymogen pro-phenoloxidase into phenoloxidase. Finally, phenoloxidase polymerizes quinones to form melanin around the pathogen and lesions [1]. Figure 5 shows that melanization begins with the formation of black or brown spots on the surface of the cuticle and can gradually spread throughout the larva’s body depending on the severity of the infection. The body of *G. mellonella* can become completely black, indicating a severe infection that generally results in the death of the larva [4].

Lysozyme, a member of the c-type lysozyme family, is an important lytic enzyme present in the hemolymph of *G. mellonella* [22,29]. Against bacteria, this enzyme hydrolyzes β-1,4 linkages. Against fungi, the enzyme acts on the cell wall, resulting in the inhibition of fungal growth [22].

## 4. Fungal Virulence Studies in *G. mellonella* Models

**Figure 6:**
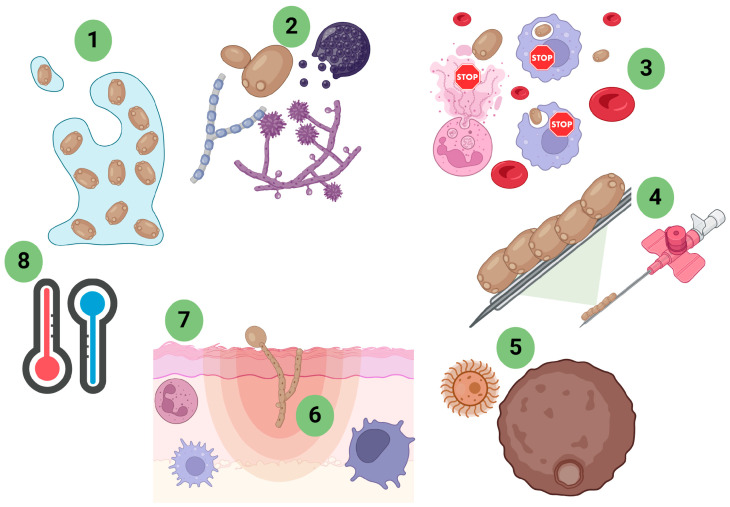
Major virulence factors utilized by fungi. (1): biofilms; (2): cellular dimorphism; (3): mediation of the immune response and survival; (4): adhesion to abiotic materials; (5) capsules or titan cells that hinder phagocytosis by the immune system; (6) melanization; (7) tissue invasion, enzyme production and inflammation; (8): thermotolerance. Created in Biorender/c41q579.

The virulence of a given fungus that causes diseases in humans may emerge from commensal or environmental factors but may also evolve during colonization and infection in the host. Factors such as heat, resistance to antifungals compounds, or resistance to the phagocytosis process may be explained by these interactions [30,31]. Figure 6 depicts several of the main virulence factors utilized by fungi that facilitate their survival in diverse hosts.

Virulence factors are highly varied. A primary requirement for infection is thermotolerance, as a fungus must be able to survive temperatures at and above 37 °C in order to cause disease in humans and other mammals. Some fungi can modify their morphology depending on the temperature, being filamentous in the environment and yeast-like at temperatures of 37 °C, called dimorphic fungi, and the different morphotypes are a challenge to the primary host response. Adhesins are extremely important for providing means for efficient attachment to tissues, which can lead to other processes such as the formation of biofilms. Structural components such as capsules enable greater survival capacity. Finally, fungi can produce enzymes such as lipases, proteinases, phospholipases, and others, which help the microbes destroy host effector cells and facilitate tissue invasion [32].

Biofilm is characterized by the presence of a community of microorganisms (single or multispecies surrounded by an extracellular matrix comprised of polysaccharides, proteins, lipids and other molecules. Components in a biofilm are largely produced by the microbe but can also incorporate materials from the host. Communities of biofilm growth microorganisms can form on both abiotic surfaces (catheter tips, stair railings, etc.) and biotic surfaces (skin, for example). Biofilms are highly sustainable and capable of hindering or preventing the penetration of drugs or recognition by host defense cells, which leads to the persistence of the infection. Therefore, biofilms are an important virulence factor for fungi [33,34,35,36].

Mycotoxins, secondary metabolic products produced by fungi, are extremely toxic to humans and animals. Poisoning by these metabolites is mainly caused by food contaminated by mycotoxins, causing changes that can lead to death. Among the main mycotoxins, aflatoxin stands out [37,38].

## 5. Virulence of Yeasts of the Genus *Candida*

*G. mellonella* have been extensively utilized to study the critically important *Candida* genus. For example, García-Carneiro et al. [39] demonstrated that *C. albicans, C. tropicalis*, *C. auris*, *C. orthopsilosis*, *C. parapsilosis*, *C. metapsilosis*, *Pichia kudriavzevii* (*C. krusei*), or *Meyerozyma guilliermondii (C. guilliermondii)* were all lethal to *G. mellonella* larvae within 6 to 10 days of infection. The study further evaluated the virulence profile of mutant and wild-type strains of *C. tropicalis* and showed that whereas the wild-type *C. tropicalis* strain killed larvae within 8 days of infection, only 50% of larvae infected with the *C. tropicalis* strains HMY175 (mnn4 Δ), HMY181 (och1 Δ), or HMY207 (pmr1 Δ) were dead by 10 days post-infection. The *C. tropicalis* mutant strains were able to stimulate greater production of hemocytes; however, the lower quantity of hemocytes in the group infected with the wild-type strain may be related to the more rapid death of the larvae. The group of larvae infected with the wild-type *C. tropicalis* showed a higher proportion of melanization and phenoloxidase production, and it was the strain with the highest toxicity index. In the same study, the authors evaluated the survival of larvae infected with clinical isolates of *C. tropicalis* and, according to the results, two distinct groups were defined: clinical isolates that killed the larvae quickly (2.2 ± 0.4 days) and clinical isolates that promoted an average survival of the larvae (5.3 ± 0.6 days). Differences were also observed in the responses of hemocytes, melanin, phenoloxidase, and cytotoxicity, and some isolates presented higher rates than others. For the other species of *Candida* evaluated in this work, the number of hemocytes was low for these species, while the production of phenoloxidase, melanin, and cytotoxicity was high in groups infected with *C. albicans*, *C. parapsilosis*, *C. orthopsilosis* or *C. auris* but low in *M. guilliermondii*, *P. kudriavzevii*, and *C. metapsilosis*.

Hernando-Ortiz et al. [40] investigated the survival profile of *G. mellonella* infected with different inoculum concentrations (105, 106, and 107 cells/larva) of *Nakaseomyces glabrata* (*C. glabrata*), *C. nivariensis*, and *C. bracarensis* and found that the mortality rate was higher as the inoculum concentration increased, with *N. glabrata* being the most virulent, followed by *C. nivariensis* and then *C. bracarensis*. The authors also reported that larvae infected with 10^7^ of *N. glabrata* presented 80% mortality by 48 h, which increased to 88% by 120 h. The group infected with *C. nivariensis* or *C. bracarensis* presented 50–60% mortality in 48 h, and mortality increased by more than 20% by 120 h. Finally, phagocytosis of *C. bracarensis* by hemocytes was more efficient when compared to the other strains.

Marcos-Zambrano et al. [41] classified the virulence profile of *Candida* sp. as low (≤30% mortality), moderate- (31−80% mortality), or high- (>80% mortality) virulence. In this study, *C. albicans* were most prevalent in the highly virulent group, with 33.3% of the total isolates, followed by *C. parapsilosis*, *C. tropicalis*, *P. kudriavzevii*, and *N. glabrata* with 24.6, 22.3, 20.8, and 8.7% of the total isolates with high virulence, respectively. The authors performed a score to evaluate the level of virulence in relation to the presence of nodules, melanization, hemocytes, and tissue invasion. According to the score with highly virulent strains, *C. albicans* most successfully invaded tissue and had a strong presence of melanization and large nodules. *C. tropicalis* also caused tissue invasion but cased necrosis, regardless of the level of virulence. *C. parapsilosis* strains generally resulted in the formation of large nodules, tissue necrosis, and melanization. *N. glabrata* caused necrosis with tissue invasion and induced high melanization. Finally, the group infected with *P. kudriavzevii* presented large nodules, but there was an absence of tissue invasion and low melanization.

Garcia-Bustos et al. [42] used the *G. mellonella* model to determine and differentiate the pathogenicity profile of different clades of *C. auris*. The results were compared, using *C. albicans* and *C. parapsilosis* as controls. *C. auris* were less virulent compared to *C. albicans* but more virulent than *C. parapsilosis*. The non-aggregative *C. auris* strains were more virulent. Furthermore, *C. auris* strains were observed to form pseudohyphae regardless of the phenotypic type (aggregative or non-aggregative). *C. auris* showed similarity to *C. albicans* in terms of its ability to disseminate. All three species showed similar melanization indices. In a later study, Garcia-Bustos et al. [43] observed that clinical isolates of *C. auris* displayed moderate degrees of larva tissue invasion but were notably more immunogenic—particularly to non-aggregative isolates—compared to the *C. albicans* and *C. parapsilosis* strains. *C. auris* also invaded the respiratory tract of the larvae, which did not occur with the other *Candida* species. Interestingly, the less-virulence-aggregative *C. auris* strains formed more filaments than the non-aggregative ones.

*G. mellonella* are a powerful tool for studying overexpression collections to define pathogenesis associated genes. For example, Pál et al. [44] used *G. mellonella* to evaluate the virulence of several prioritized *C. parapsilosis* mutants in comparison to wild-type (genes *CPAR2_100460* to *CPAR2_806950*). The investigators found that three strains were significantly more lethal compared to the control, resulting in approximately a 10% survival for the mutants compared to 75% survival for the wild-type strain. In contrast, one mutant was attenuated compared to the wild type and did not induce larval melanization. These results clarified the association of the genetic targets with virulence in *C. parapsilosis*. The mutant strain *CPAR2_107240*, one of the most virulent strains, presents orthology with the CaKTR4/MNT4 gene. This ortholog is verified as a mannosyltranferase in *C. albicans* and is associated with N-linked glycosylation and cell wall regeneration

## 6. Virulence of Non-*Candida* Yeasts

Torres et al. [45] aimed to use the *G. mellonella* model to establish an in vivo model for *Malassezia furfur* and *Malassezia pachydermatis* infection. Both yeasts caused systemic infection in the larvae. Virulence was related to temperature, species and inoculum size. *M. pachydermatis* was slightly more virulent at 37 °C than *M. furfur*. The survival rate was dependent on the inoculum concentration, with higher inoculum concentrations resulting in higher mortality rates for both yeasts. Melanization also generally increased except for a reduction in the melanin score in the largest inoculum injected. Additionally, larvae infected with *M. furfur* at 33 °C had a higher melanin score than those incubated at 37 °C. While plasmocytes increased as yeast concentration increased, other hemocytes decreased for both yeasts. Finally, histological tests showed the formation of granulomas, which were more present at higher inoculum concentrations. *M. furfur* granulomas were found more frequently in the inoculation region. In the larvae infected with *M. pachydermatis*, the granulomas were more disseminated.

Garcia-Rodas et al. [46] used the *G. mellonella* model to investigate the morphological response of *Cryptococcus neoformans*. To obtain cells with larger capsule size, 10% Sabouraud medium buffered at pH 7.3 with 50 mM MOPS was used. Infection led to an increase in capsule size, which was more pronounced at 37 °C incubation, resulting in greater virulence and reduced phagocytosis. The authors link the increase in the capsule to the greater difficulty in phagocytosis by the hemocytes. Giant cells recovered from mice caused larval mortality similar to cells cultured in vitro models. Trevijano-Contador and Zaragoza [47] evaluated the immune response profile of *G. mellonella* against *C. neoformans*. *G. mellonella* presented high lytic activity in the hemolymph, being dependent on the presence of the capsule and temperature. *C. neoformans* caused intracellular infection of hematocytes. Furthermore, *C. neoformans* did not cause melanization, while the control *C. albicans* yeasts did induce pigmentation. The virulent profile of biofilms and planktonic cells of *C. neoformans* and *C. gatti* were evaluated by Benaducci et al. [48]. Larvae infected with biofilms growth fungi of either species were rapidly killed, with all larvae dead at 4 days post-infection. In contrast, when infected with planktonic cells of *C. gatti* and *C. neoformans*, mortalities were 70 and 20% on Day 4 of infection, respectively.

## 7. Virulence of Dimorphic Fungal Pathogens in *G. mellonella*

Clavijo-Giraldo et al. [49] utilized *G. mellonella* to assess the virulence profile *of Sporothrix schenckii* sensu stricto and *Sporothrix brasiliensis*. They tested low-, intermediate-, and high-virulence strains according to the murine model and found that larval survival was dependent on the concentration of conidia, germilings, or yeasts of *S. brasiliensis* and *S. schenckii*. *S. brasiliensis* was more virulent that *S. schenckii* strains. Lozoya-Perez et al. [50] reported the importance of the method of preparing strains of *S. schenckii*, *S. brasiliensis*, and *S. globose* on outcomes of larval infection. The authors cultivated the strains in different media, including yeast–peptone–dextrose, Sabouraud broth, potato-dextrose broth, and brain–heart infusion. Carbon and nitrogen limitation reduced the virulence of *S. schenckii* and *S. brasiliensis* but did not significantly affect the lethality of *S. globosa*. *S. brasiliensis* and *S. schenckii* were more virulent when cultivated in yeast–peptone–dextrose medium. Finally, all strains were less virulent in the *G. mellonella* model when the yeasts were cultivated in Sabouraud or potato-dextrose broth. Reis et al. [51] also evaluated the virulent profile of *S. brasiliensis* in *G. mellonella*. Over the course of infection, there was an increase in the fungal load as well as in the number of hemocytes with larval death occurring as early as day 4 post-infection. The death of the larvae was observed from the 4th day after infection.

Scorzoni et al. [52] also used a *G. mellonella* model to evaluate the virulence of *Paracoccidioides brasiliensis* and *Paracoccidioides lutzii* and found that mortality and phagocytosis rates were similar between the strains studied, and both species reduced the number of hemocytes. However, *P. lutzii* had more interactions with hematocytes. Similar to the findings in the study above by Thomas et al. [51], the study also measured a higher expression of glycoprotein g43 in P. lutzii, which may be linked to the greater adhesion with hemocytes. Scorzoni et al. 2018 [53] subsequently demonstrated that *Paracoccidioides* spp. lost virulence in wax moths after successive subcultures. However, virulence could be recovered after passage in mammals. The authors observed that the *G. mellonella* model was able to reactivate the adhesion of *P. brasiliensis*, important in the virulence of the fungus, as well as faster isolation of the yeast when compared to another in vivo model, mice.

Marcos et al. [54] used *G. mellonella* to evaluate the importance of the TufM protein (previously known as EF-Tu) in *P. brasiliensis*. They found that a downregulated strain of TufM with a 55% reduction in expression, obtained by antisense RNA, significantly reduced the ability of the fungus to kill both *G. mellonella* and mice. The percentage of larvae alive after 7 days of being infected with the down-regulated strain was 52.7%, while all larvae were dead by 5 days after infection with the wild-type strain, confirming the importance of this protein in virulence of *P. brasiliensis*.

Paracoccin is an important glycoprotein produced by *P. brasiliensis* roles in fungal biology and pathogenesis. Pitangui et al. [55] evaluated the profile of *P. brasiliensis* overexpressing paracoccin, wild-type strain, and paracoccin-downregulated strain in a wax moth infection model. The overexpression strain was the most virulent, as infection resulted in 25% larval survival at 12 days, whereas the wild type and the down-regulated strain had survival rates of 37.5 and 67.5%, respectively. These findings recapitulated prior findings in mice [56].

Barker et al. [57] injected different *Coccidioides posadassi* spores into *G. mellonella* to evaluate virulence with a focus on melanization. The studies showed that larval death and melanization were dependent on the concentration of injected spores. The lethal dose (LD50) was between 4 and 6 × 10^5^ spores of *C. posadasii*, and the LD100 was between 0.5 and 1.0 × 10^6^ spores. The melanization score peaked after 2 days of infection. The presence of nodules was observed via histology, and arthroconidia converting into fragments of hyphae and spherules were also visualized, which demonstrated that the fungus can convert and complete its life cycle within the hemocoel of the larva. Garcia et al. [58] also observed that increasing the inoculum of *C. podassi* increased the mortality rate of the larvae. Although infections with 10^4^ spherules produced survival curves similar to the group treated with PBS, increasing the infectious dose to 10^6^ spherules/larva led to a 100% mortality by 4 days post-infection. A 10^6^ inoculum with heat-killed spherules also decreased larval survival.

In addition to investigating the virulence of *P. lutzii*, Thomaz et al. [59] infected *G. mellonella* to determine the virulence of *Histoplasma capsulatum* at an environmental and human body temperature. *H. capsulatum* decreased larval survival at both temperatures; however, infections at 37 °C resulted in higher mortality rates. Additionally, lethality was directly proportional to inoculum concentration. At the highest concentration, 10^6^ yeasts/larva, all larvae died by 15 days at 37 °C. However, at 25 °C, no concentration tested caused 100% mortality. Blocking the main *H. capsulatum* adhesin for macrophages, heat shock protein (Hsp) 60, with a specific antibody resulted in a marked attenuation of virulence in a wax moth infection study [60]. Although infection with G186A and EH-315 strains caused 78 and 73.5% mortality rates, respectively, blocking of Hsp60 resulted in approximately only 40% mortality for both strains.

*Penicillium marneffei* virulence was studied in a *G. mellonella* larvae at 25 and 37 °C [61]. Mortality was directly proportional to the concentration of injected fungus and the larvae had more severe disease at 37 °C. For example, on the 4th day of infection with 10^5^ CFU wild-type conidia/larva), 50 and 30% of the larvae survived when incubated at 25 or 37 °C, respectively. Inoculums of 10^3^, 10^5^, and 10^7^ CFU/larva were 100% lethal all larvae infected with 10^7^ yeasts died after 24 h (for both temperatures). After 4 days, 30 and 50% of the larvae infected with 10^5^ cells survived when incubated at 37 and 25 °C, respectively. Furthermore, conidia were observed inside hemocytes after 2 h of infection, and the number of internalized fungal cells increased over the course of the experiment. After 4 days of infection, larvae infected with a wild-type strain were completely melanized. Finally, the authors also observed a difference in pathogenicity between two strains of *P. marneffei*. All larvae infected (1 × 10^5^ CFU/larva at 25 °C) with the red-pigment-producing strain died within 4 days, while the group similarly infected with the non-pigment-producing strain all survived during this period of time.

## 8. Other Fungi That Cause Opportunistic Infections

Mauer et al. [62] used a *G. mellonella* model to evaluate the pathogenic profile of *Aspergillus terreus* strains resistant or susceptible to amphotericin B. Mortality rates were directly proportional to the inoculum size and temperature. All larvae infected with 10^7^ conidia/larva at 37 °C died by 72 h, while at 30 °C mortality was 70% during this interval. The strains susceptible to amphotericin B were more virulent compared to the resistant strains. The virulent potential was based on the mean survival time, which was 60–72 h and 96–108 h for the amphotericin B-susceptible and -resistant strains, respectively. Won et al. [63] also evaluated the virulence of clinical isolates of *A. terreus*, isolated from the respiratory tract or ear. The results showed that there was a variation among strains in their virulence at 72 h, but there was no significant difference in relation to the isolated site. In total, 31 clinical isolates were evaluated. The average survival of the larvae was 32.6% (72 h after inoculation), with a range from 5% to 72.5%.

Bakti et al. [64] described the effect of the PIB-type cation ATPase PcaA, which links metal homeostasis and heavy metal tolerance in *A. fumigatus* in a *G. mellonella* model using PcaA deletion, PcaA overexpression, gfp-PcaA complementation strain, and wild-type strains. The survival of larvae infected with the PcaA mutant strain was higher than that of those infected with the wild-type strain. The mortality of strains overexpressing pcaA was higher than that of the group infected with the wild-type strain, which strengthens the importance of PcaA in the virulence of *A. fumigatus*.

To explore the role of phenoloxidase and melanization in *Aspergillus niger*, Staczek et al. [65] injected *A. niger* α-1,3-glucan into *G. mellonella* to determine the possible inhibition completely of phenoloxidase activity in the initial phase. Transient enzyme appearance was detected 2–8 h and 1–6 h with α 1,3-glucan and conidia inoculation. Maximum detected enzyme activity was reached after 4 h of challenge when challenged with α 1,3-glucan. However, when challenged with conidia, the enzyme level remained low. Finally, enzyme activity began to decrease after 6 h, being completely decreased after 24 h when challenged with α 1,3-glucan. In this fungus, decreased phenoloxidase levels resulted in a less robust immune system response by larvae and consequently increased the effectiveness of the infection.

Curtis, Walshe, and Kavanagh [66] infected larvae with a wild-type *A. fumigatus* control and compared this to *A. fumigatus* subjected to 25 passages on Galleria extract agar. Cultivation on Galleria agar led to attenuation of the fungus. For example, in 48 h of testing, the mortality of larvae infected with control strains was 30% compared to larvae infected with past strains with a mortality of 5–8%. *A. fumigatus* cultivated on Galleria agar were also less susceptible to killing by hemocytes, with a mortality rate of 9.19 to 41.37% in 40 min compared to 92.8% with the non-passaged fungus in the same time interval.

Li et al. [67] evaluated the effects of putative *A. fumigatus* Ca^2+^-binding sites of calmodulin, which is involved in morphogenesis and pathogenicity, using a *G. mellonella* infection model. In situ alterations to four Ca^2+^-binding sites of calmodulin reduced the virulence of the mutants. The data indicate that the mutant strain caused a mortality of 25% within eight days of infection, significantly less virulent than the parental wild strain. All larvae infected with the wild strain died within eight days of infection. Hence, the four Ca^2+^-binding sites are involved in the virulence of *A. fumigatus*.

Rudhra et al. [68] used non-melanized and melanized *A. flavus* strains to observe the role of the pigment in an infection model using *G. mellonella*. Melanized conidia (clinical isolate) were more virulent than non-melanized conidia, supporting the importance of melanin as a virulence factor in this species. Additionally, non-melanized conidia were more susceptible to killing by hemocytes, and the authors suggest that antigenic surface proteins are masked by melanin, making it difficult for immune cells to recognize them, resulting in attenuation of the host response.

To study the role of sirtuin-signaling proteins, Wassano et al. [69] infected larvae with six single-mutation strains of *A. fumigatus*. All mutant strains were hypervirulent, killing all larvae between Days 9 and 13, with the exception of the ∆sirE strains. The strain with mutations in all sirtuins was less virulent than the group infected with the wild-type strain. Given this, it is observed that sirtuins play an important role in the virulence of *A. fumigatus* in a non-vertebrate model.

Other studies have also used *G. mellonella* models to evaluate the virulence profile of *A. fumigatus* [70,71,72,73], *A. leporis* [74], *A. flavus* [75,76,77,78], and others, but we have insufficient space to address these interesting works. *A. fumigatus* strains with triple mutation (G641/G643/E664) had their virulence significantly reduced when compared to double-mutation strains (G641/G643), which caused mortality similar to the non-mutated group (wild) 70. Deletion of Afrtt109 reduced the virulence of *A. fumigatus* 71. Mutant strains ΔgprM and ΔgprJ had their virulence reduced 73. However, selection of ArgAN or ArgB increased their virulence 72. Another study observed that decreasing lysergic acid α-hydroxyethylamide, a specialized metabolite, reduced the virility of *A. leporis* 74. *A. flavus* strains were not more virulent than other non-pathogenic strains, *A. parasiticus*, *A. arachidicola*, and *A. nomiae* 75. The importance of the enzyme phosphomannose isomerase for *A. flavus* was studied and, according to the data, the mutants presented attenuated virulence 76. The hexokinase enzyme AfHxk1 played a profound role in the pathogenicity of *A. flavus* in *G. mellonella* 77. Finally, resistant strains of *A. flavus* showed reduced virulence 78.

Navarro-Velasco et al. [79] studied the importance of the Rapamycin complex (TORC1)—a regulator of growth and development—in *Fusarium oxysporum*, a fungus that causes diseases in plants and immunocompromised humans in a *G. mellonella* infection model. They noted that *F. oxysporum* strains expressing the green fluorescent protein (GFP) gene developed hyphae in the hemolymph and interacted with hemocytes. Significantly, they found that mutating the TORC1 negative relator Tuberous Sclerosis Complex 2 (Tsc2) attenuated the fungus, as the tsc2Δ mutant had a reduced ability to invade larval tissue, adherent hyphae formation, and decreased virulence in *G. mellonella*. The mortality rates of larvae infected with the wild-type strain and complemented tsc2Δ were 100% at 3 and 5 days of infection, while the tsc2Δ strain failed to kill all larvae during the experiment. Another study investigated the importance of zinc mediated by *F. oxysporum* ZafA, a transcription factor, in the wax moth [80]. ZafA deletion strains had significantly reduced tissue invasion capacity and lower larval mortality rates.

Maurer et al. [81] utilized models of *G. mellonella* to investigate fungal species responsible for mucormycosis, including *Rhizopus* spp., *Rhizomucor* spp., *Lichtheimia* spp. and *Mucor* spp. In general, virulence was specific for each species, and inoculum concentration, incubation temperature, iron availability, and stress tolerance were important variables in the outcomes of the interactions of the fungi with *G. mellonella*. For example, *Rhizopus* spp. killed all larvae at 24 h and was the most virulent in the *G. mellonella* model. Larval mortality due to infection with these fungi was reduced at the lower temperature, 30 °C.

Hassan et al. [82] used a wax moth model to study the role of the outer layer of *Lichtheimia corymbifera* sporangiospores in virulence. The authors used *L. corymbifera* and *L. corymbifera* subjected to different physical and chemical treatments. Combined glucano-proteolytic treatments were required to attenuate fungal virulence in the wax moth. This treatment resulted in increased phagocytosis, a 10-fold increase in phagolysosomal fusion after fungal uptake, and significant extensions in larval survival after infection. Liang et al. [83] used *L. corymbifera*, *M. irregularis*, *M. hiemalis*, and *R. arrhizus* to evaluate their virulence profile in *G. mellonella*. Interestingly, this study showed that the pathogenicity of Mucoralis was related to energy metabolism, with mitochondria playing an important role in energy production. Mortality rates were highly variable for these strains as *R. arrhizus* and *L. corymbifera*, killed 90 and 20% of infected larvae by 96 h, whereas *M. irregularis* and *M. hiemalis* did not kill larvae during the timeframe of the experiment.

Spore coat proteins (CotH) are present in bacteria and fungi and play an important role in pathogenesis. Disruption of the *Mucor lusitanicus* cotH4 gene caused a decrease in ability of the fungus to kill *G. mellonella*, which was confirmed in both murine and *Drophophila* models [84]. Interestingly, deletion of cotH3 attenuated virulence in the fly and mouse models but not in *G. mellonella*, underscoring the challenges with broad extrapolations from infection models. Thanapaul et al. [85] also observed that the morphology of mucoralis species impacts their virulence. They found that infection with *R. arrhizus* and *L. corymbifera* hyphae caused approximately 10–16-times-higher mortality rates than the group infected with sporangiospores, with a significant reduction in the lethal dose (LD50) of hyphae for *R. arrhizus* and *L. corymbifera*. *L. corymbifera* was the most virulent strain for both the hyphae form and sporangiospores. In five days of infection, the group infected with hyphae presented survival of 10, 60 and 100% for the inocula 2.68 × 10^3^, 1.34 × 10^2^, and 2.50 × 10^1^, respectively. The group infected with conidia presented survival of 25, 60, 90, and 100% for the inocula 1.88 × 10^4^, 1.85 × 10^3^, 1.45 × 10^2^, and 2.25 × 10^1^, respectively.

Fuchs et al. [86] examined the capacity of *Pneumocystis murina* to cause disease in *G. mellonela*. Interesting, the fungus did not cause death or melanization of larvae, even at 216 h.

Chromoblastomycosis or chromomycosis are fungal infections caused by different species of dematiaceous (pigmented) fungi [86]. Liu et al. [87] used a *G. mellonella* model to study melanin producing and albino strains of *Fonsecaea monophora*. Interestingly, the albino mutant strains caused greater pathogenicity and produced larger inflammatory nodules. In contrast to what was previously discussed with this pigment in other fungi, Fonsecaea melanin was associated with a more robust immune response in the larva. *Cladophialophora carrionii* is also a pathogen that causes chromoblastomycosis, and Shi et al. [88] investigated the virulence of the fungus in *G. mellonella*. Virulence depended on the injected dose, growth rate, and melanization of the fungus. The strain *C. carrionii* CBS 131736, an environmental strain, presented higher mortality rates, which were associated with a higher melanization rate and higher growth rate. Another environmental strain, *C. carrionii* CBS 861.96, was non-virulent, and it grew slowly and had a low melanization rate. Histology assays showed the presence of nodules, with dimensions and numbers increasing depending on the biological characteristics of the strain. The most notable presence of nodules was for groups infected with *C. carrionii* CBS 17900 and CBS 114406, which were also highly virulent strains.

Mycetoma is a subcutaneous mycosis caused by different fungi and is known for forming white or black granules in the tissues [89]. One of the genera causing mycetoma is *Madurella* sp. and Kloezen et al. [90] observed the formation of *M. mycetomatis* granules in larvae, which were similar to those found in lesions of humans and mammals. Two strains tested, strains mm55 and cn796, caused the death of all larvae after 7 and 8 days, respectively, with an inoculum of 4 mg of wet weight per larva.

*G. mellonella* infection models can be used also for dermatophytes, albeit with variable results between experiments [91,92]. In one series of experiments, investigators found that injection or rolling of larvae with dermatophytes did not cause significant mortality, but cultivation on dermatophyte-covered plates, particularly using YEPD medium, resulted in significant mortality of the insects [91]. More than 80% of larvae infected with 106 conidia of *Microsporum canis*, *Trichophyton tonsurans* and *T. rubrum* survived. However, when larvae were directly incubated on the “dermatophyte lawn” (plet tracking method), differences between species were observed. In this method, the authors reported some problems, such as the growth of yeast and bacteria, which may have interfered with the mortality of the larvae. The method with Sabouraud agar was more plausible. The effect of mating type locus on virulence has also been explored in the wax moth model. *M. gypseum* strains with (−) and (+) mating types were found to have similar rates of killing, which suggests that mating type may not significantly impact virulence in this species [92].

**Table 1:** Studies involving fungal virulence assays in *Galleria mellonella* models.

Fungi	Size or Weight of Larva	Inoculum	Quantity	Ref.
*Candida* spp.	1.2–1.5 cm	2 × 10^7^ cells/larva	10 µL	[39]
*N. glabrata*, *C. nivariensis* and *C. bracarensis*	0.3–0.5 g	1 × 10^5^, 1 × 10^6^ and 1 × 10^7^ cells/larva	10 µL	[40]
*Candida* sp.	330 ± 20 mg	5 × 10^5^ cells/larva (*C. albicans* and *C. tropicalis*) and 2 × 10^6^ 5 × 10^6^ cells/larva (*C. parapsilosis*, *N. glabrata*, and *P. kudriavzevii* )	10 µL	[41]
*C. auris, C. albicans* and *C. parapsilosis*	250 to 350 mg	10^5^ and 10^6^ CFU/Larva	10 µL	[42]
*C. auris, C. albicans* and *C. parapsilosis*	250 to 350 mg	10^6^ CFU/Larva	10 µL	[43]
*C. parapsilosis*	-	5 × 10^5^ cells/larva	10 µL	[44]
*M. furfur* and *M. pachydermatis*	250 and 330 mg	range 1.5 × 10^6^–1.5 × 10^9^ CFU/mL)	20 μL	[45]
*C. neoformans*	-	10^4^ cells/larva	10 μL	[46]
*C. neoformans* and *C. albicans*	200–300 mg	10^4^ to 10^6^ cells/larva	10 μL	[47]
*Cryptococcus neoformans var. grubii*	100–200 mg	5 × 10^6^ cells/larva	10 μL	[48]
*S. schenckii* sensu stricto and *S. brasiliensis*	1 cm	1 × 10^5^, 1 × 10^6^ or 1 × 10^7^ cells/μL	10 μL	[49]
*S. schenckii* sensu stricto, *S. brasiliensis* and *S. globosa*	1.2–1.5 cm	1 × 10^5^ yeast-like cells	10 μL	[50]
*S. schenckii* sensu stricto and *S. brasiliensis*	0.2–0.3 g	1 × 10^7^ cells/larva	10 μL	[51]
*P. brasiliensis* and *P. lutzii.*	150–200 mg	5 × 10^5^, 1 × 10^6^ and 5 × 10^6^ cells/larva)	-	[52]
*P. brasiliensis*		5 × 10^6^ cells/larvae		[53]
*P. brasiliensis*	01–0.2 g	5 × 10^6^ cells/larvae	10 μL	[54]
*P. brasiliensis*	150–200 mg	5 × 10^6^ cells/larva	10 μL	[55]
*C. podassi*	0.2–0.4 g	10^3^ to 10^6^ spores	10 μL	[57]
*C. podassi*	245 mg +/− 25 mg	10^4^ and 10^6^ total spherules/larva	8 μL	[58]
*H. capsulatum*	0.1–0.15 g	(10^1^, 10^2^, 10^3^, 10^4^, 10^5^ and 10^6^ cells/larva	10 μL	[59]
*H. capsulatum*	150–200 mg	1 × 10^6^ yeasts/larvae	10 μL	[60]
*P. marneffei*	300–350 mg	10^1^, 10^3^, 10^5^ and 10^6^ CFU/Larva	10 μL	[61]
*A. terreus*	0.3–0.4 g	1 × 10^5^, 1 × 10^6^ and 1 × 10^7^ conidia/larva	20 μL	[62]
*A. Terreus*	~150 mg	-	10–50 μL	[63]
*A. fumigatus*	-	8 × 10^4^ conidios	20 μL	[64]
*A. niger*	-	1 × 10^5^/larva	3 μL	[65]
*A. fumigatus*	0.2–0.3 g	5 × 10^5^ conidia	20 μL	[66]
*A. fumigatus*	0.3 g	-	10 μL	[67]
*A. flavus*	160–200 mg	10^3^ germinated conidia	10 μL	[68]
*A. fumigatus*	330 mg	-	10 μL	[69]
*A. fumigatus*	-	1 × 10^8^	10 μL	[70]
*A. fumigatus*	-	1 × 10^8^ spore	-	[71]
*A. fumigatus*	0.4 and 0.5 g	1 × 10^7^ freshly harvested	20 μL	[72]
*A. fumigatus*	275 to 330 mg	1 × 10^6^/larva	5 μL	[73]
*A. leporis*, *A. hancockii*, and *A. homomorphus*	-	3000 conidia/μL or, in one experiment, 250 conidia/μL	20 μL	[74]
*A. flavus*	~300 mg	1 × 10^6^, 10^4^ and 10^3^/larva	5 μL	[75]
*A. flavus*	-	10^4^ conidia	-	[76]
*A. flavus*	-	10^7^/mL	5 μL	[77]
*A. flavus*	-	-	-	[78]
*F. oxysporum*	0.2–0.3 g	1.6 × 10^3^ microconidial/larva	8 μL	[79]
*F. oxysporum*	-	-	-	[80]
*Rhizopus* spp., *Rhizomucor* spp., *Lichtheimia* spp., *Mucor* spp.	0.3–0.4 g	1 × 10^4^, 1 × 10^5^, 1 × 10^6^ and 1 × 10^7^ conídios/larva	20 μL	[81]
*L. corymbifera*	0.3–0.4 g	10^7^ spores /larva	20 μL	[82]
*M. irregularis*, *M. hiemalis*, *L. corymbifera* and *R. arrhizus*	-	1 × 10 ^5^ esporos/larva	10 μL	[83]
*M. lusitanicus*		10^5^ cells/larva	20 μL	[84]
*R. arrhizus* and *L. corymbifera*	250 ± 50 mg	10^1^ to 10^6^ sporangiospores or germinated sporangiospores	10 μL	[85]
*Pneumocystis murina*	330 ± 25 mg	4.85 × 10^5^ or 4.85 × 10^6^ cells per larvae	10 μL	[86]
*F. monophora*	250–300 mg	10^4^, 10^5^ and 10^6^ conidia /larva	20 μL	[87]
* C. carrionii *	300–500 mg	10^4^, 10^5^ and 10^6^ conidia /larva	40 μL	[88]
*M. mycetomatis*	300–500 mg	0.04 to 4 mg wet weight per larvae	40 μL	[90]
*M. gypseum, M. canis*, *T. rubrum*, *T. mentagrophytes*, *T. equinium* and *T. tonsurans*	0.3–0.4 g	10 ^6^ conídios ml ^ −1 ^ In medium	5 μL	[91]
*M. gypseum, M. canis*, *T. rubrum*, *T. mentagrophytes*, *T. equinium* and *T. tonsurans*	-	-	-	[92]

## 9. Analysis of the Studies Involved

**Figure 7:**
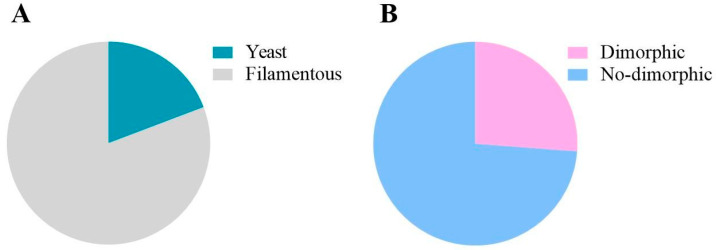
Analysis of fungal strains used in in vivo studies using *Galleria mellonella*. (**A**) Analysis of studies involving filamentous fungi or yeasts; (**B**) Analysis of studies involving dimorphic or non-dimorphic fungi.

Figure 7 presents data from studies involving yeast-like or filamentous fungi (Figure 7A). Furthermore, among the filamentous fungi, there are dimorphic and non-dimorphic ones (Figure 7B). According to the analysis, 19.2% of the studies evaluated the yeast profile in G. mellonella, while 80.8% involved filamentous fungi. Of the total filamentous fungi, 26.2% were with dimorphic strains and 73.8% involved non-dimorphic fungi.

**Figure 8:**
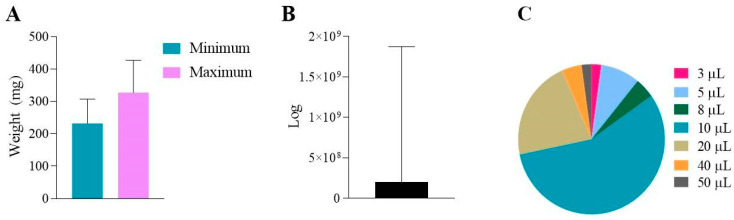
Methodological analysis of studies using the *Galleria mellonella* model. (**A**) analysis of variation in minimum and maximum weight of larvae used in the methodology; (**B**) average amount (with standard deviation) of inoculum inserted into the larvae; (**C**) Amount of solution inserted into larvae (in µL).

According to the studies, the average minimum and maximum weight of the larvae was 232 and 327, respectively (Figure 8A). Inoculums ranged from 10^1^ to 10^9^ cells, with an average of 1.99 × 10^8^ cells (Figure 8B). Finally, more than half of the studies, 56.5%, injected 10 µL of solution (treatment or inoculum) into *G. mellonella*. A total of 21.7% used 20 µL. The other 8.7, 4.3, 4.3, 2.2, and 2.2% were with 5, 8, 40, 50, and 3 µL, respectively (Figure 8C).

## 10. Drugs Available or Under Investigation with Future Antifungal Application

In the scientific environment, future drugs are evaluated in in vitro assays, and then the best candidates are evaluated in in vivo studies, including *G. mellonella* models [1]. In addition to the similarities between the immune system and mammalian cells, other factors found in *G. mellonella* are advantageous for using this model in antimicrobial assays, such as ease of inoculation, low cost, and rapid results (24–48 h). The low cost allows experiments to be performed in high replicates and in a short time, generating greater reliability in the data quickly. Furthermore, larvae are easy to handle and do not require authorization from an ethics organization to perform the assays. Finally, as they can be cultivated at a temperature similar to that of the human body, they thus allow the evaluation of the antimicrobial action of pathogens that cause infection in humans, including fungi. Therefore, the number of investigations using this model as a promising in vivo assay to evaluate therapeutic effects continues to grow [93,94]. Table 2 summarizes the selected studies with fungi covered in this review that used drugs available or under investigation in *G. mellonella*.

Miltefosine is a drug licensed for the treatment of leishmaniasis and breast cancer, however, it has shown considerable antifungal activity. For example, encapsulated miltefosine in an alginate nanoparticle has been evaluated in a wax moth infection model using larvae infected with *C. albicans, Cryptococcus neoformans* or *C. gattii*. The larvae infected with *C. albicans* or *C. gattii* and treated with encapsulated miltefosine exhibited prolongation of their survival. Additionally, reductions in fungal load were observed in both plaque samples and histological assays [95]. Barreto et al. [96] subsequently evaluated the efficacy of miltefosine against *C. auris*. In vitro results showed that miltefosine killed *C. auris*, and treatment with free or encapsulated miltefosine improved the survival of infected larvae. Furthermore, there was a significant reduction in the fungal load and the number of granulomas when compared to the untreated group. Subsequently, the authors evaluated the effects against *A. fumigatus* and *A. flavus*, including using the drug with or without voriconazole. The in vivo results showed that miltefosine had efficacy against the molds and that there was a synergistic effect between the drugs, since there was a greater reduction in the larval mortality rate and fungal load in the larvae receiving combination therapy. Therefore, either miltefosine alone or encapsulated and combined with voriconazole could be an important strategy in therapy against aspergillosis [97] and other fungi.

Statins are substances used to control cholesterol levels in the bloodstream and prevent cardiovascular accidents. However, they have an effect against infectious diseases, including directly inhibiting the growth of diverse microorganisms [98]. Atorvastatin, a statin, was investigated by Ajdidi et al. [99] against *C. albicans* using *G. mellonella*. Atorvastatin increased the survival rate of infected larvae. While infected larvae without treatment had a survival rate of 18.1 ± 4.2% at 144 h, the group treated with atorvastatin had a survival of 60.2 ± 6.4%.

Miramistin is an important antiseptic, and its antifungal potential was evaluated by Osmanov et al. [100] against *C. albicans* and *A. fumigatus* in a wax moth model. In *G. mellonella*, miramistin (16 mg kg^−1^ and 160 mg kg^−1^) was protective against infection at certain concentrations of both fungi. However, miramistin did not prevent either infection at a concentration of 1000 mg/kg^−1^, possibly due to cumulative toxicity.

Acetylcholine is synthesized by human cells and acts by modulating fungal virulence as well as regulating the immune response. Given this, Nile et al. [101] evaluated the potential of pilocarpine hydrochloride to control *C. albicans* infection in *G. mellonella*. Pilocarpine inhibited *C. albicans* lethality by preventing filament formation and regulating the larval immune response. Furthermore, acetylcholine and pilocarpine hydrochloride modulated the hemocyte response to *C. albicans*. The muscarinic receptor was found to regulate filamentation and biofilm formation by *C. albicans*. Furthermore, hemocyte subsets have a repertoire of cholinergic receptors that are capable of regulating differentiation, function, and activation.

Additional drugs in clinical use or pre-clinical development have been examined in wax moth studies. For example, fenbendazole, MMV1782387, ravuconazole, and olorofilm were shown to prolong the lifespan of *G. mellonella* infected with *M. mycetomatis*. After ten days of infection and treatment, the survival rates of infected larvae treated with olorofilm, MMV1782387, ravuconazole, or fenbendazole were 33.3, 28.9, 26.7, and 24.4%, respectively [102].

Manogepix is an active moiety of the prodrug fosmanogepix, a first-in-class antifungal in phase II clinical trials. Interestingly, manogepix failed to increase the survival of *G. mellonella* infected with *M. mycetomatis* and treated at 8.6 mg/kg of the drug. Furthermore, the combination of manogepix with itraconazole did not increase survival. The authors concluded that there was a limited therapeutic potential of manogepix against *M. mycetomatis*, which causes mycetoma [103].

Other drugs—such as rezafungin, encochleated amphotericin B, oteseconazole, opelconazole, ibrexafungerp, and fosmanogepix—are among the new antifungals recently released or in advanced development [104]. However, no studies involving these drugs in *G. mellonella* models have been published to date.

## 11. Antifungal Combinations Available to Fight Infection

The worrying incidence of infection is an increasing concern in the clinical setting, especially as monotherapy, in some cases, is not efficient. Combined therapies are being explored to enhance our capacity to combat diverse lethal fungal diseases. The objective of combination approaches includes improving therapeutic efficacy and concomitantly reducing mortality rates [105]. We discuss select studies using combined therapies against diverse fungi in *G. mellonella* models.

Linozoline, a synthetic oxazolidinone, was combined with different azoles, fluconazole, itraconazole and voriconazole, and evaluated in *G. mellonella* infected with *C. albicans*. The combination of linozoline with azoles was synergistic since it prolonged the life of the larvae up to two times when compared to monotherapy. In addition, synergism was also shown by the decrease in fungal loads [106]. Similarly, when fluconazole (160 μg/mL) was combined with D-penicillamine (40 μg/mL) against *C. albicans*, there was an improvement in antifungal activity, with greater survival of infected larvae and a decrease in fungal load (in 4 days assay) [107].

Verapamil is an important efflux pump inhibitor, and Vega-Chacón et al. [108] evaluated the combination of verapamil and fluconazole against fluconazole-resistant *C. albicans*. The combination showed synergism with reduced larval mortality (*p* ≤ 0.012) as well as reduced fungal recovery. However, there was no difference between the group treated with fluconazole alone and the group treated with saline. A synergistic effect was also observed between fluconazole and eravacycline, synthetic halogenated tetracycline, since an increase in the survival rate of *G. mellonella* infected with *C. albicans* resistant to fluconazole was observed [109]. Xiuyun Li et al. [110] also combined fluconazole with teriflunomide, an immunosuppressant, and observed a synergistic effect in *G. mellonella* infected with *C. albicans*. The survival rates for the group treated with fluconazole or teriflunomide alone, on Days 2 and 4 of infection, were 35–70% and 33–69%, respectively. However, when treatment was given in combination, the survival rates on Days 2 and 4 of infection were 69 and 87%, respectively.

Combination therapy with liposomal amphotericin B and flucytosine followed by fluconazole and flucytosine is currently standard for the genus *Cryptococcus* [111]. However, mortality rates remain high, and additional combinations are being explored. Several studies have shown additional synergistic combination regimens against *C. neoformans* using *G. mellonella* models, inlcuding the combination of pedalitin, a tetrahydroxy-monohydroxy-flavone, and amphotericin B [112]; ethyl acetate extract of poincianella pluviosa queiros stem bark with amphotericin B [113]; itraconazole with hydroxychloroquine [114]; minocycline with fluconazole [115]; and pyrvinium pamoate, an inhibitor of mitochondrial functions and a Wnt inhibitor combined with either itraconazole or fluconazole or posoconazole or amphotericin B or voriconazole [116].

The TOR pathway, a target of rapamycin, acts in the regulation of lipid homeostasis, autophagy, ribosome biogenesis, translation, transcription, and other important activities of the fungus, being fundamental for the pathogenicity and virulence. Yi Sun et al. [117] evaluated the TOR inhibitor AZD8055 in combination with azoles against different genera of fungi, including *Candida* sp, in a wax moth model. The results showed efficacy, where TOR AZD8055 combined with azoles increased the survival of *G. mellonella* infected with *C. albicans* and *C. auris*. The survival rates of larvae infected with *C. auris* and treated with AZD8055 with posaconazole, AZD8055 with itraconazole, and AZD8055 with voriconazole were 41.7%, 40%, and 48.3%, respectively. With the group infected with *C. albicans*, the survival of larvae treated with AZD8055 with posaconazole, AZD8055 with itraconazole, AZD8055 with voriconazole and AZD8055 with fluconazole were 48, 45, 66.7, and 41.7%, respectively. Important action was also observed in *A. fumigatus*, *E. dermatitidis* and *C. neoformans*.

Drug combinations have also shown effecacy in inhibiting *Aspergillus* sp. infection in *G. mellonella* models. Studies have shown synergism between voriconazole and terbinafine. At 8 days of infection with *A. calidoustus*, markedly improved survival was observed compared to the untreated group [118]. The TOR inhibitor AZD8055 in combination with azoles was synergism. The survival of larvae infected with *A. fumigatus* and treated with the combination of AZD8055 with POS, AZD8055 with itraconazole, and AZD8055 with voriconazole were 50%, 46.7%, and 58.3%, respectively. [117]. The combination of miltefosine + voriconazole was evaluated in *G. mellonella* infected with *A. fumigatus*. The survival rate ranged from 70–78% when compared to 40% for monotherapy against. There was no difference between miltefosine nanoparticle + voriconazole compared to monotherapy [97]. Other studies evaluated minocycline combined with voriconazole, itraconazole, or posoconazole. The survival rates of groups infected with *A. fumigatus* and treated with minocycline with voriconazole, minocycline with itraconazole, and minocycline with posaconazole were 53.3, 43.3, and 58.3%, respectively [119].

*G. mellonella* infected with *Fusarium* spp. also showed prolonged survival when treated with chlorhexidine and voriconazole or natamycin. While monotherapy with chlorhexidine presented a survival rate of 10%, the combination of chlorhexidine with voriconazole was 33% [120]. The synergistic effect between the combination of minocycline combined with voriconazole, itraconazole or posoconazole was tested against *F. solani*. The survival rates of larvae infected with *F. solani* and treated with minocycline with voriconazole, minocycline with itraconazole, and minocycline with posaconazole were 46.7, 15, and 21.7%, respectively [119].

Combination therapies against *Rhizopus microspores* have been explored in wax moth models. For example, the combination of voriconazole with amphotericin B, posaconazole or caspofungin was tested against *R. microspores* [121]. The best combination was voriconazole with amphotericin B, which resulted in greater larval survival. Furthermore, another important result was that the combination resulted in therapeutic efficacy with a 32-fold lower dose of amphotericin B when compared with monotherapy. In this same study, the authors found that species-specific differences existed as the combinations produced highly disparate results in different mucormycosis species. Notably and strikingly, another study showed that the combination of amphotericin B with terbinafine or itraconazole caused a higher mortality rate of *G. mellonella* infected with *M. mycetomatis* [122]. Hence, combinations must be carefully examined as they do not all result in a synergistic effect and may even be harmful.

*G. mellonella* have been used to explore the efficacy of novel compounds. For example, alkaloids present in the venom of the fire ant (*Solenopsis invicata*) were recently tested against *C. auris*, and both natural and synthetic sole opsins inhibited the in vitro growth of *C. auris* strains from different clades, including fluconazole- and amphotericin-B-resistant isolates. Doses of sole opsin up to 50 µg/mL were not toxic in *G. mellonella*. Although infection studies were not performed, the *G. mellonella* toxicity results together with the in vitro data suggest that these compounds be further explored for combatting *C. auris* and other fungi [123].

**Table 2:** Drugs available or under investigation with future antifungal application tested in *Galleria mellonella* models.

Drugs Candidate	Fungi	Dose/Larva	Ref.
Miltefosine i	*C. albicans*, *C. neoformans* and *C. gattii*	0.03–16 µg/mL miltefosine	[95]
Miltefosine	*C. auris*	20 or 40 mg/kg of miltefosine free and 100 mg/kg miltefosine encapsulated	[96]
Miltefosine alone or Voriconazole combinate	* A. fumigatus * and *A. flavus*	20 or 40 mg/kg of miltefosine free, 100 mg/kg miltefosine encapsulated, 20 mg/kg of miltefosine + 10 mg/Kg of voriconazole free, and 100 mg/kg miltefosine encapsulated with 10 mg/Kg voriconazole	[97]
Atorvastatin	*C. albicans*	4.55 or 9.09 mg/kg^−1^	[99]
Miramistin	*C. albicans* and *A. fumigatus*	16, 160 and 1000 mg/kg^−1^	[100]
Pilocarpine hydrochloride and acetylcholine	*C. albicans*	3.12, 6.25 and 10.5 mM	[101]
Miconazole, ravuconazole, oteseconazole, eberconazole, luliconazole, fenbendazole, carbendazim, amorolfine, tafenoquine, alexidine olorofilm and others	*M. mycetomatis*	20 μM of compound per larvae	[102]
Manogepix	*M. mycetomatis*	8.57 mg/kg manogepix and 5.71 mg/kg itraconazole	[103]
Azoles + linozoline	*C. albicans*	FLC (160 μg/mL), ITZ (40 μg/mL), VRC (40 μg/mL), LZD (200 μg/mL), LZD (200 μg/mL) + FLC (160 μg/mL), LZD (200 μg/mL) + ITZ (40 μg/mL), and LZD (200 μg/mL) + VRC (40 μg/mL),	[106]
Fluconazole and D-penicillamine	*C. albicans*	D-penicillamine 40 (μg/mL) and FLU (160 μg/mL)	[107]
Verapamil and fluconazole	*C. albicans*		[108]
Eravacycline and fluconazole	*C. albicans*		[109]
Teriflunomide and Fluconazole	*C. albicans*	ERV (2 μg/larva) + FLC (1 μg/larva)	[110]
Liposomal amphotericin B and flucytosine	*Cryptococcus* sp.		[111]
Pedalitin and amphotericin B	*C. neoformans*	AmB 0.3 mg/kg + PED 10 mg/kg	[112]
ethyl acetate extract of *poincianella pluviosa* with amphotericin B	*C. neoformans*	2 MIC and 4 MIC (EAF/AmB: 3.9/0.003, 7.8/0.006, and 15.6/0.015 g/mL, respectively, for two strains)	[113]
hydroxychloroquine and itraconazole	*C. neoformans*	3 mg/kg ITR, and 6.5 mg/kg HCQ	[114]
Minocycline and Fluconazole	*C. neoformans*	5.2 mg/kg for the FLU and 6.3 mg/kg MINO	[115]
Pyrvinium pamoate, fluconazole, itraconazole, voriconazole, posaconazole or amphotericin B	*C. neoformans*	200 mg/L	[116]
TOR inhibitor AZD8055, Itraconazole, Voriconazole, fluconazole and Posoconazole	*Candida* sp., *C. neoformans, Aspergillus* and *E. dermatitidis*	-	[117]
Voriconazole and terbinafine	*A. calidoustus*	-	[118]
Miltefosine alone or Voriconazole combinate	* A. fumigatus * and *A. flavus*	20 or 40 mg/kg of miltefosine free, 100 mg/kg miltefosine encapsulated, 20 mg/kg of miltefosine + 10 mg/Kg of voriconazole free, and 100 mg/kg miltefosine encapsulated with 10 mg/Kg voriconazole	[97]
Minocycline and Itraconazole, voriconazole or posoconazole	*Aspergillus* sp., *Fusarium* sp. and *E. dermatitidis*	-	[119]
Chlorhexidine and voriconazole or natamycin	*Fusarium* sp.	VOR (3 μg/mL), CHL (1.5 μg/mL), and VOR + CHL (3 μg/mL and 1.5 μg/mL, respectively).	[120]
Coriconazole (plus amphotericin B, posaconazole and caspofungin	*R. microspores*, *R. oryzae*, *Syncephalastrum racemosum*, *Lichthemia corymbifera*, *L. blakesleeana*, and *L. ramosa*,	1 mg/kg for AMB and CSF and 10 mg/kg for VRC and PSC	[121]
Amphotericin B and Terbinefine or itraconazole	*M. mycetomatis*	1 mg/kg of AmB, 5.7 mg/kg ITZ, and 7.14 mg/kg of terbinafine	[122]
Alkaloids solenopsins	*C. auris*	0.5, 5 and 50 µg/mL of synthetic mixture of solenopsins or natural mixture solenopsins	[123]

FLC: Fluconazole, ITZ: Itraconazole; LZD: Linozoline; VRC: Voriconazole; AmB: Amphotericin B; PSC: Posoconazole; CSF: Caspofungin; ERV: Eravacyclin; HCQ: Hydroxychloroquine; MINO: Minocycline; PED: Pedalitin; EAF: ethyl acetate extract of poincianella pluviosa.

## 12. Nanomaterials as Promising Vehicles in the Future

Nanostructured systems can be considered a promising therapy for the future since they improve the efficacy and reduce toxicity of a given drug against a pathogen and contribute to the control of various infections [124]. Table 3 summarizes select studies that explore the efficacy of nanomaterials using *G. mellonella* models.

Liposome (LP) is a vesicle composed of a lipid bilayer, one of the most widely used lipid nanomaterials for hydrophilic and hydrophobic drugs [125]. Vera-González et al. [126] developed an encapsulated LP with anidulafungin to increase its solubility and antifungal activity against *C. albicans*. The liposomes increased the activity of anidulafungin in infected *G. mellonella* larvae. Depending on the liposome used, the survival rate was from 33% to 67% within 7 days of infection compared to 25% survival for the group treated with the conventional drug. However, another study observed that LP encapsulated with amphotericin B did not increase the survival of larvae infected with *Rhizopus* spp., *Rhizomucor* spp., or *Mucor* spp., except for *L. corymbifera* (30% better compared to the untreated group during 144 h post-infection). According to the authors, the low efficacy of antifungals for the *G. mellonella* model is related to the pharmacokinetics of the antifungals [81]. In contrast, LP encapsulated with amphotericin B was effective against fluconazole-resistant *C. parapsilosis* and multidrug-resistant *C. parapsilosis* [127]. The study was compared with groups treated with micafungin and fluconazole and, according to the results, liposomal amphotericin increased the survival rate against all strains tested in *G. mellonella*, except for CP70 (*p* = 0.545) and CP207 (*p* = 0.359).

EFG1 is an important regulator of filamentous morphology and virulence of *C. albicans*, and Araújo et al. [128] developed LPs composed of cationic dioleoyl-trimethylammoniumpropane to encapsulate EFG1 for use in treatment of *C. albicans*. In a *G. mellonella* model, EFG1 LPs were more effective, increasing larval survival by 45% at 48 h and 25% at 72 h of testing.

Nanoemulsions (NE) are emulsions formed by two immiscible liquids, water and oil, and, with the help of surfactants, it is possible to form droplets of water in oil or oil in water. NEs are kinetically stable [129]. Marena et al. [94] developed an NE loaded with amphotericin B and evaluated its anti-*Candidozyma auris* profile in a wax moth infection model. The NE enhanced the antifungal activity of AmB with a significant reduction in the fungal load. The difference in fungal load between nanoemulsion with amphotericin B and amphotericin B alone was approximately more than one log on the last day of infection (5 days of treatment). Subsequently, the authors developed an NE loaded with micafungin and the results were also promising since the action of micafungin loaded in NE was better than micafungin alone in the *C. auris G. mellonella* model. In this study, although conventional micafungin was efficient, with the eradication of fungal load in 3 days of treatment, the NE loaded with micafungin anticipated the eradication of fungal load in hemolymph, with the absence of growth in 2 days of treatment (out of a total of 5 days) [33]. Additional *C. auris* clades were subsequently tested in this model with positive results, suggesting that NE can be considered a promising vehicle for the delivery of micafungin and amphotericin B against different clades of *C. auris* [19,130].

Nanoparticles have been explored to expand the range of molecules for clinical use, including solid lipid nanoparticles (SLNs) and nanostructured lipid carriers (NLCs). These two lipid nanomaterials can carry chemically distinct drugs and provide tunable release [131]. Passos et al. [132] developed an NLC loaded with itraconazole for topical application against *S. brasiliensis* and *C. albicans*. The results with *G. mellonella* showed that the group treated with NLC had a higher survival rate, indicating that NLC better the antifungal action of itraconazole. NLC with itraconazole protected all larvae, with 100% survival (20–40 mg/kg). However, conventional itraconazole did not preserve larval survival and health.

Incorporation of chitosan into a nanoparticle (Nq) enhances several properties of the particle, such as better fixation of particles in tissue, increasing contact time with cells and improving the immune response [5]. Costa et al. [133] incorporated farnesol in Nq and investigated the possible interference of the nanomaterial in *G. mellonella* infected with *C. albicans*. Nq with farnesol increased larval survival and significantly acted in reducing fungal load and inhibited the formation of hyphae and biofilms.

Metal nanoparticles (Mn), including zinc Mn, have been studied due to their antimicrobial potential, mainly due to their membrane data, biomolecule induction and induction of reactive oxygen species. Nian-Xu et al. [134] developed a zinc oxide Mn to determine its anti-*Candida albicans* potential in a *G. mellonella* model. The zinc oxide MN increased larval survival and decreased fungal load. After 8 days, 67% and 27% of larvae infected with *C. albicans* died when pretreated with PBS solution and amphotericin B, respectively. However, when treated with zinc oxide in Mn, viability was higher when compared to the PBS group. At a 0.2 mg/kg concentration of zinc oxide in Mn, mortality was reduced by 13% compared to the PBS group. However, Mn was relatively toxic at low concentrations (≤20 mg/kg).

Thammasit et al. [135] developed a nanocarrier, poly (n-butyl cyanoacrylate) nanoparticles containing propolis, a resin-like material made by bees, to facilitate the particle crossing the blood-brain barrier for the purpose of reducing the infection and virulence of *C. neoformans.* A *G. mellonella* model showed that therapy with the new nanocarrier increased the survival of infected larvae. The infected and untreated larvae presented melanized bodies, with 80% death after 4 days of infection (all died after 7 days). The group that received the treatment presented a survival rate of approximately 90% after 4 days of infection.

Poly(lactic-co-glycolic) acid (PLGA) is a biopolymer widely used in the development of nanomaterials for drug delivery, and Orekhova et al. [136] incorporated pterostilbene, a 3,5-dimethylated derivative of resveratrol, into a PLGA nanoparticle and evaluated the antifungal profile in *G. mellonella* infected with *A. brasiliensis*. The results showed that the group infected with the nanomaterial incorporated with pterostilbene presented a significant dose-dependent reduction in mortality when compared to the group treated with free pterostilbene.

**Figure 9:**
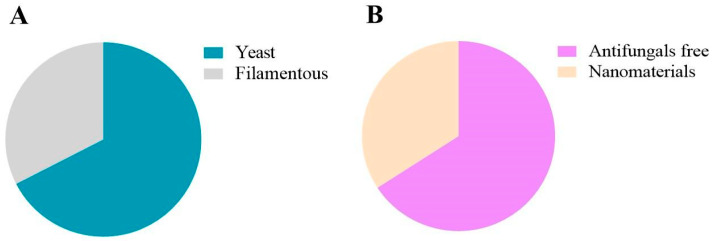
Methodological analysis of studies that evaluated the antifungal profile of future antifungal options. (**A**) therapy against filamentous fungi or yeasts; (**B**) therapy or nanotherapy.

Finally, of the total studies that evaluated therapeutic models, 67.4% were against infections caused by yeasts while 32.6% evaluated the antifungal profile using filamentous fungi (Figure 9A). Of the future therapeutic options, 65.9% are from materials not involving nanomaterials while 34.1% come from nanomaterials (Figure 9B).

**Table 3:** Studies involving the development of nanomaterials for drug incorporation and evaluation with in vivo *Galleria mellonella* models.

Nanomaterial	Fungi	Drug	Ref.
AN	*C. albicans* and *Cryptococcus* sp.	0.78–600 μg/mL miltefosine encapsulated	[95]
LP	*C. albicans*	Anidulafungin	[126]
LP	*Rhizopus* spp., *Rhizomucor* spp., *Mucor* spp., and *Lichtheimia* spp.	Amphotericin B	[80]
LP	*C. parapsilosis*	Amphotericin B	[127]
LP	*C. albicans*	EFG1	[128]
NE	*C. auris*	Amphotericin B	[94]
NE	*C. auris*	Micafungin	[33]
NE	*C. auris*, *C. albicans* and *C. parapsilosis*	Amphotericin B	[19]
NE	*C. auris*, *C. albicans* and *C. parapsilosis*	Micafungin	[130]
NLC	*S. schenckii* and *C. albicans*	Itraconazole	[132]
Nq	*C. albicans*	Farnesol	[133]
Mn	*C. albicans*	Zinc oxide Mn	[134]
Nanocarreador	*C. neoformans*	Propolis	[135]
Nanoparticles PLGA	*A. brasiliensis*	Pterostilbene	[136]

AN: Alginate nanoparticles; LP: liposome; SLN: solid lipid nanoparticles; NLC: nanostructured lipid carriers; Mn: metal nanoparticles; Nq: chitosan nanoparticles; PLGA: poly(lactic-co-glycolic) acid.

## 13. Conclusions

As fungal infections increase, new treatment options are needed, especially in the face of systemic and opportunistic fungal infections, including emerging mycoses. With the pandemic caused by SARS-CoV-2 and the associated increase in diverse mycoses together with the increasing reports of drug resistance in myriad fungi, this need has become even more necessary and urgent. The use of alternative in vivo models to evaluate the infection profile and antimicrobial action has increased in recent years and this has contributed to the discovery, characterization, and validation of new and promising antimicrobials. The *G. mellonella* model has become one of the most widely used models today in the scientific field, serving as an auxiliary method in determining pathogenicity, virulence, antifungal activity, immunogenicity, and toxicity, among others. Therefore, as the present work clearly shows, the *G. mellonella* method can be considered a promising in vivo model for studies involving different fungal pathogens and contribute to investigating new antifungal therapies.

The *G. mellonella* model may present a good alternative for the recovery of virulence of fungal strains, since the microorganisms are exposed to conditions not found in culture media or in vitro assays, with the immune response being one of the main factors. Furthermore, the *G. mellonella* model is considered reliable, and its results can be compared to those of studies involving other living beings, such as models involving the parasite *Caenorhabditis elegans*, the fly *Drosophila melanogaster*, and tests with mice among others. By using these alternative tests, it is possible to reduce the use of animals, such as mice, in certain experiments without interfering with the reliability and coherence of the data, reaching a plausible conclusion. On the other hand, there are restrictions on the use of this model, such as the fact that it is not a mammalian model, which distances it somewhat from the results that can be found in other in vivo models. The weight of the larvae can also present significant variation, which can interfere with the final results. Different maintenance and handling methodologies are found in the literature, which also interferes with the interpretation of data between different studies that used the same strains.

These studies are a source of hope for global health and could provide significant advances in the discovery of new antifungal therapies, contribute to controlling the spread of human pathogens, and impact the reduction of fungal diseases. This is the main objective since the population lacks new treatment alternatives, especially against fungal pathogens.

## Data Availability

Data are contained within the article.
